# 

**Published:** 1894-06-30

**Authors:** 


### CONTENTS.

A Physician to Physicians - - 265

Around the Hospitals - - 266

Medical Progress and Hospital Clinics.—On Some Manifestations of Scorbutic Disease in Children - - 273

Diseases of the Ear - - 274

MEDICAL PROGRESS.—Typhoid (*continued*) - - 275

PROGRESS IN SURGERY.—Liver and Gall Bladder Surgery of Pancreas and Spleen - - 277

Malignant Growths - - 278

NEW APPLIANCES AND THINGS MEDICAL - - 279

The First Century of English Literature.—VI. The Library of the Chirurgeon - - 267

Some Aspects of Medical Science - - 268

The Study of Man.—VII. Language and Race - - 269

The Field of Science - - 270

Annotations.—Guardians and Nurse Gillespies—A "Bitter Cry" from Cornwall—Dry Sanitation - - 271

Notes and News - - 272

Editor's Letter-Box - - 280

The Institutional Workshop:

THE STORY OF THE INSANE FROM YEAR TO YEAR - - 281

HOSPITAL CONSTRUCTION.—The Homœopathic Hospital, Great Ormond Street - - 282

PRACTICAL POINTS 282; HOSPITAL FESTIVALS AND MEETINGS - 284

### EXTRA SUPPLEMENT.—THE NURSING MIRROR.

News from the Nursing World.—Errors of Diet—Mistaken Identity—Good Work Done Well—At the Albert Hall—"A Fad of the Lady Guardians"—An Excess of Examinations—The Rights of the Poor—What is "The Plague?"—At Detroit—Good Tidings—Short Items - - cxxii

On General Nursing.—XVII. Peritonitis - - cxxiii

English Nurses in Brazil.—Rio de Janeiro - - cxxiv

Minor Appointments - - cxxv

Notes from Germany - - cxxv

For Reading to the Sick - - cxxvi

Craig Colony for Epileptics - - cxxvi

Exeter City Asylum - - cxxvi

The Mechanism of Movement.—VI. The Spinal Column - cxxvii

A Case for Assistance - - cxxvii

Wants and Workers - - cxxvii

Two Hours Off Duty - - cxxviii

Sentence Passed on "Nurse" Gillespie - - cxxviii

Novelties for Nurses - - cxxviii

The Muses' Looking Glass - - cxxix

Notes and Queries - - cxxix

Everybody's Opinion—Where to Go - - cxxx

Queen's Nurse at Seacombe - - cxxx

